# Phylogenetic analysis of erythritol catabolic loci within the *Rhizobiales* and Proteobacteria

**DOI:** 10.1186/1471-2180-13-46

**Published:** 2013-02-23

**Authors:** Barney A Geddes, Georg Hausner, Ivan J Oresnik

**Affiliations:** 1 Department of Microbiology, University of Manitoba, R3T 2N2, Winnipeg, MB, Canada

## Abstract

**Background:**

The ability to use erythritol as a sole carbon source is not universal among the Rhizobiaceae. Based on the relatedness to the catabolic genes in *Brucella* it has been suggested that the *eryABCD* operon may have been horizontally transferred into *Rhizobium*. During work characterizing a locus necessary for the transport and catabolism of erythritol, adonitol and L-arabitol in *Sinorhizobium meliloti,* we became interested in the differences between the erythritol loci of *S. meliloti* and *R. leguminosarum.* Utilizing the Ortholog Neighborhood Viewer from the DOE Joint Genome Institute database it appeared that loci for erythritol and polyol utilization had distinct arrangements that suggested these loci may have undergone genetic rearrangements.

**Results:**

A data set was established of genetic loci containing erythritol/polyol orthologs for 19 different proteobacterial species. These loci were analyzed for genetic content and arrangement of genes associated with erythritol, adonitol and L-arabitol catabolism. Phylogenetic trees were constructed for core erythritol catabolic genes and contrasted with the species phylogeny. Additionally, phylogenetic trees were constructed for genes that showed differences in arrangement among the putative erythritol loci in these species.

**Conclusions:**

Three distinct erythritol/polyol loci arrangements have been identified that reflect metabolic need or specialization. Comparison of the phylogenetic trees of core erythritol catabolic genes with species phylogeny provides evidence that is consistent with these loci having been horizontally transferred from the alpha-proteobacteria into both the beta and gamma-proteobacteria. ABC transporters within these loci adopt 2 unique genetic arrangements, and although biological data suggests they are functional erythritol transporters, phylogenetic analysis suggests they may not be orthologs and probably should be considered analogs. Finally, evidence for the presence of paralogs, and xenologs of erythritol catabolic genes in some of the genomes included in the analysis is provided.

## Background

Operons are multigene arrangements transcribed as a single mRNA and are one of the defining features found in bacterial and archaeal genomes. This arrangement allows genes to be co-regulated, and members of operons are usually involved in the same functional pathway [1,2]. Although operons are prominent features in the genomes of bacteria and archaea, the evolution and mechanisms that promote operon formation are still not resolved and a number mechanisms have been proposed [3-8]. These mechanisms involve dynamic genetic events that include gene transfer events, deletions, duplications, and recombinations [2,5,8]. Since operons are prominent features in bacterial genomes, and often encode genes with metabolic potential, it may be assumed that their evolution is under some selection pressure, thus allowing prokaryotic cells to rapidly adapt, compete and grow under changing environmental conditions.

The metabolic capability of an organism can be a function of its genome size and gene complement and these greatly affect its ability to live in diverse environments. The alpha subdivision of the proteobacteria includes some organisms that are very similar phylogenetically but inhabit many diverse ecological niches, including a number of bacteria that can interact with eukaryotic hosts [9]. The genome sizes of these organisms varies from about 1 MB for members of the genus *Rickettsia* to approximately 9 MB for members of the bradyrhizobia [10]. Comparative genomic studies of this group has led to the supposition that there has been two independent reductions in genomic size, one which gave rise to the *Brucella* and *Bartonella*, the other which gave rise to the *Rickettsia*[11]. In addition, it also suggests that there has been a major genomic expansion and that roughly correlates with the soil microbes within the order Rhizobiales [11]. The genomes of Rhizobia are dynamic. Phylogenetic analysis of 26 different *Sinorhizobium* and *Bradyrhizobium* genomes recently showed that recombination has dominated the evolution of the core genome in these organisms, and that vertically transmitted genes were rare compared with genes with a history of recombination and lateral gene transfer [12]. In this manuscript we have utilized comparative genomics in a focused manner to investigate the evolution of genes and loci involved in the catabolism of the sugar alcohols erythritol, adonitol and L-arabitol, primarily within the alpha-proteobacteria.

The number of bacterial species that are capable of utilizing the common 4 carbon polyol, erythritol, as a carbon source is restricted [13]. Catabolism of erythritol has been shown to be important for competition for nodule occupancy in *Rhizobium leguminosarum* as well as for virulence in the animal pathogen *Brucella suis*[14]. Genetic characterization of erythritol catabolic loci has only been performed in *R. leguminosarum*, *B. abortus* and *Sinorhizobium meliloti.* In these organ-isms erythritol is broken down to dihydroxyacetone-phosphate using the core erythritol catabolic genes *eryABC-tpiB*[15]. During characterization of the erythritol locus of *S. meliloti*, it was observed that despite the close homologies of core erythritol genes, the genetic content and arrangement of the locus was drastically different from the previously characterized loci of *B. abortus* and *R. leguminosarum*[16]. In particular the locus encodes the catabolism of two 5-carbon pentitols (adonitol and L-arabitol) in addition to erythritol. It was shown that the ABC transporter encoded by *mptABCDE* and erythritol kinase encoded by *eryA* can also be used for adonitol and L-arabitol, and several genes in the locus are involved in adonitol and L-arabitol, but not erythritol catabolism including *lalA-rbtABC*[15].

The differences between the erythritol loci in the sequenced *S. meliloti* strain Rm1021 [17], and *R. leguminosarum*, led us to question what the relationship of these erythritol catabolic loci may be to other putative erythritol catabolic loci in bacterial species. In this work we focus on this question by analyzing the content and synteny of loci containing homologs to the erythritol genes in other sequenced organisms. The results of the analysis lend support to several hypotheses regarding operon evolution, and in addition, the data predicts loci that may be involved in polyol transport and metabolism in other proteobacteria.

## Methods

### Identification of erythritol loci

The data set of erythritol loci utilized in this work was constructed in a two-step process. First BLASTN was used to identify sequenced genomes containing homologs to the core erythritol catabolic genes *R. leguminosarum* and *S. meliloti*[18]. The use of BLASTN rather than BLASTP at this stage allowed us to refine the search to bacteria with sequenced genomes. Furthermore, limiting the search to genes with highly similar sequences by using BLASTN allowed us to limit our search to only genes that are likely involved in erythritol catabolism, since all of these genes encode proteins in highly ubiquitous families found throughout bacterial genomes. Initially BLASTN searches were performed using all the core erythritol genes shared between *R. leguminosarum* and *S. meliloti* (*eryA, eryB, eryC* and *eryD*). However, the search using *eryA* provided the most diverse data set that also showed a sharp drop in E-value and query coverage. Using either *eryA* from *R. leguminosarum*, or *eryA* from *S. meliloti* for the BLASTN search resulted in an identical data set. Genomes containing homologs to *eryA* were selected on the basis of E-values less than 1.00E-5. In cases where multiple strains of the same bacterial species were found to have highly homologous putative erythritol genes (>99% identity) only a single representative of the species was used to avoid redundancy. Additionally *B. melitensis* 16M and *B. suis* 1330 were chosen as representatives of the *Brucella* lineage despite a large number of *Brucella* species that were identified in our search due to the high degrees of similarity between their erythritol catabolic genes.

Second, the genetic region containing *eryA* in these organisms was identified and analyzed using the IMG Ortholog Neighborhood Viewer (http://img.jgi.doe.gov) [19] in order to construct the gene maps (loci). The amino acid sequence of EryA from *S. meliloti* was used as a query for the IMG Ortholog Neighborhood Viewer search.

To analyze the genetic content of organisms in our data set, the amino acid sequence encoded by each gene involved in erythritol catabolism in *R. leguminosarum*, or in erythritol, adonitol or L-arabitol catabolism in *S. meliloti*, was individually used in a BLASTP search of the 19 genomes in the data set. The sugar binding proteins of the *S. meliloti* and *R. leguminosarum* transporter were used as representatives of the entire ABC transporter. Identity cut-off values that were used to delineate potential homologs to erythritol proteins were unique to each query amino acid sequence. Cut-off values were as follows: MptA: 56%, EryD: 44%, EryA: 46%, RbtA: 50%, EryB: 65%, LalA: 49%, RbtB: 51%, RbtC: 40%, EryC: 68%, TpiB: 69%, EryR: 61%, EryG: 73%. These values were manually determined and generally correlated to a large drop in percentage identity within the BLASTP hits.

Homologs identified that were not within the primary *eryA* containing loci were used as a query within IMG-Ortholog neighborhood viewer to analyze the region surrounding them. Secondary loci containing homologs to some of these genes were identified in *Mesorhizobium* sp. and *Sinorhizobium fredii*. These loci are putative erythritol loci based on homology to known loci involved in erythritol catabolism in *Sinorhizobium meliloti*[15,16]*, Rhizobium leguminosarum*[20]*and Brucella abortus*[21]. Despite not having been experimentally verified we will refer to all loci in our data set as erythritol loci for the purpose of this manuscript.

### Phylogenetic analysis

Amino acid sequences of homologs to proteins previously shown to play a role in erythritol, adonitol or L-arabitol catabolism from each of the organisms in the data set were collected and used for phylogenetic analysis. The 16S *rDNA* and RpoD sequences were also extracted from the NCBI database for species examined in this study in order to obtain a potential species tree that could be compared with the various phylogenetic gene trees obtained from the individual genes located within the polyol (i.e. erythritol, arabitol, and adonitol) utilization loci. Amino acid sequences were aligned using Clustal-X [22] and PRALINE [23] the resulting alignments were refined manually with the GeneDoc program v2.5.010 [24].

Phylogenies were generated with maximum likelihood analysis (ML) as implemented in the Molecular Evolutionary Genetic Analysis package (MEGA5) [25] and with MrBayes [26]. MEGA5 was used to identify the most suitable substitution models for the aligned data sets. In order to evaluate support for the nodes observed in the ML phylogenetic trees bootstrap analysis [27] was conducted by analysing 1000 pseudo replicates.

The MrBayes program (v3.1) was used for Bayesian analysis [26,28] and the parameters set for amino acid alignments were mixed models and for the 16S rDNA gamma distribution with 4 rate categories. The models used (setting mixed model) for generating the final 50% majority rule trees were estimated by the program itself. The Bayesian inference of phylogenies was initiated from a random starting tree and four chains were run simultaneously for 1 000 000 generations; trees were sampled every 100 generations. The first 25% of trees generated were discarded (“burn-in”) and the remaining trees were used to compute the posterior probability values.

Phylogenetic trees were constructed for RpoD, 16S rDNA and all the key genes associated with the EryA genes. Phylogenetic trees were plotted with the TreeView program [29] using MEGA5 and/or MrBayes tree outfiles. Final trees were annotated using Adobe Illustrator.

## Results

### Phylogenetic distribution of putative erythritol loci

Based on homology to *eryA* from *Sinorhizobium meliloti* and *Rhizobium leguminosarum* we have compiled a data set of 19 different putative erythritol loci from 19 different proteobacteria (Table  1). Previous studies suggested that erythritol loci may be restricted to the alpha-proteobacteria [20]. While a majority of the erythritol loci we identified followed this scheme, surprisingly we identified putative erythritol catabolic loci in *Verminephrobacter eiseniae* (a beta-proteobacterium) and *Escherichia fergusonii* (a gamma-proteobacterium). Erythritol loci are not widely distributed through the alpha-proteobacteria. A majority of the loci we identified were within the order Rhizobiales. Outside of the Rhizobiales we also identified erythritol loci in *Acidiphilium* species and *Roseobacter* species. Within the Rhizobiales, erythritol loci were notably absent from a large number of bacterial species such as *Rhizobium etli, Agrobacterium tumefaciens* and *Bradyrhizobium japonicum* that are closely related to other species that we have identified that contain erythritol loci. We also note that erythritol loci appear to be plasmid localized only in *S. fredii* and *R. leguminosarum*. In all other cases the loci appear to be found on chromosomes.

**Table 1:** Bacterial genomes used in this study containing erythritol loci

**Genome**	**Accession number**	**Reference/ Affiliation**
*Sinorhizobium meliloti* 1021	AL591688.1	[17]
*Sinorhizobium medicae* WSM419	CP000738.1	[30]
*Sinorhizobium fredii* NGR234	CP000874.1	[31]
*Mesorhizobium opportunism* WSM2075	CP002279.1	US DOE Joint Genome Institute
*Mesorhizobium loti* MAFF303099	BA000012.4	[32]
*Mesorhizobium ciceri* bv. *biserrulae* WSM1271	CP002447.1	US DOE Joint Genome Institute
*Bradyrhizobium* sp. BTAi1	CP000494.1	[33]
*Bradyrhizobium* sp. ORS278	CU234118.1	[33]
*Agrobacterium radiobacter* K84	CP000629.1	[34]
*Ochrobactrum anthropi* ATCC 49188	CP000759.1	[35]
*Brucella suis* 1330	CP002998.1	[36]
*Brucella melitensis* 16M	AE008918.1	[37]
*Acidiphilium multivorum* AIU301	AP012035.1	NITE Bioresource Information Center
*Acidiphilium cryptum* JF-5	CP000697.1	US DOE Joint Genome Institute
*Roseobacter denitrificans* Och114	CP000362.1	[38]
*Roseobacter litoralis* Och149	CP002623.1	[39]
*Rhizobium leguminosarum* bv. viciae 3814	AM236086.1	[40]
*Rhizobium leguminosarum* bv. trifolii WSM1325	CP001623.1	[41]
*Verminephrobacter eiseniae* EF01-2	CP000542.1	US DOE Joint Genome Institute
*Escherichia fergusonii* ATCC 35469	CU928158.2	Genoscope - Centre National de Sequencage

### Genetic content of loci

**Figure 1:**
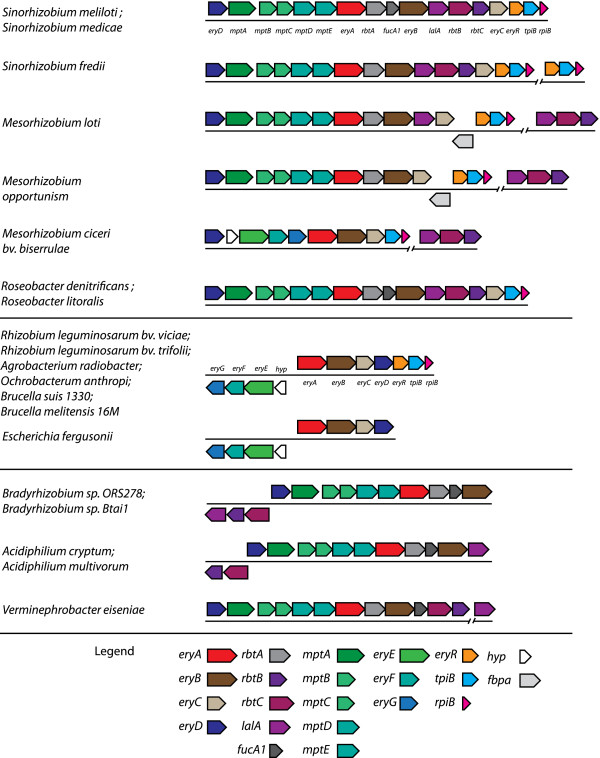
**The genetic arrangement of putative erythritol loci in the proteobacteria.** Genes are represented by coloured boxes and identical colours identify genes that are believed to be homologous. Gene names are given below the boxes for *Sinorhizobium meliloti* and *Rhizobium leguminosarum.* Loci arrangements are depicted based on the output from the IMG Ortholog Neighborhood Viewer primarily using the amino acid sequence EryA from *Sinorhizobium meliloti*, and *Rhizobium leguminosarum*. Gene names in the legend generally correspond to the annotations in *R. leguminosarum* and *S. meliloti*.

The genetic content of each of the organisms *ery* loci were analyzed by conducting a BLASTP search to the 19 genomes in our data set of the amino acid sequence of each gene associated with erythritol catabolism in *R. leguminosarum*, or erythritol, adonitol or L-arabitol catabolism in *S. meliloti.* The results of the BLAST search are presented in Table  2, depicting the presence or absence of homologs to erythritol, adonitol or L-arabitol catabolic genes in each of the genomes that was investigated. Gene maps of erythritol loci were constructed based on the output of our IMG Ortholog Neighborhood Viewer searches and are depicted in Figure  1.

**Table 2:** Content of putative erythritol loci

**Genome**	**Homologs involved in erythritol, adonitol and/or L-arabitol catabolism**											
	**EryA**	**EryB**	**EryD**	**EryC**	**EryG**	**EryR**	**TpiB**	**MptA**	**LalA**	**RbtA**	**RbtB**	**RbtC**
*Sinorhizobium meliloti*	+	+	+	+	-	+	+	+	+	+	+	+
*Sinorhizobium medicae*	+	+	+	+	-	+	+	+	+	+	+	+
*Sinorhizobium fredii*	+	+	+	+	-	++	++	+	+	+	+	+
*Mesorhizobium opportunism*	+	+	+	+	-	+	+	+	+	+	+	+
*Mesorhizobium loti*	+	+	+	+	-	+	+	+	++	+	+	+
*Mesorhizobium ciceri* bv. *biserrulae*	+	+	+	+	+	-	+	-	+	-	+	+
*Roseobacter denitrificans*	+	+	+	+	-	-	+	+	+	+	+	+
*Roseobacter litoralis*	+	+	+	+	-	-	+	+	+	+	+	+
*Rhizobium leguminosarum* bv. *viciae*	+	+	+	+	+	+	+	-	-	-	-	-
*Rhizobium leguminosarum* bv. *trifolii*	+	+	+	+	+	+	+	-	-	-	-	-
*Agrobacterium radiobacter*	+	+	+	+	+	+	+	-	-	-	-	-
*Ochrobacterum anthropi*	+	+	+	+	+	+	+	-	-	-	-	-
*Brucella suis* 1330	+	+	+	+	+	+	+	-	-	-	-	-
*Brucella melitensis* 16M	+	+	+	+	+	+	+	-	-	-	-	-
*Escherichia fergusonii*	+	+	+	+	+	-	-	-	-	-	-	-
*Bradyrhizobium* sp. BTAi1	+	+	+	-	-	-	-	+	+	+	+	+
*Bradyrhizobium* sp. ORS278	+	+	+	-	-	-	-	+	+	+	+	+
*Acidiphilium multivorum*	+	+	+	-	-	-	-	+	+	+	+	+
*Acidiphilium cryptum*	+	+	+	-	-	-	-	+	+	+	+	+
*Verminephrobacter eiseniae*	+	+	+	-	-	-	-	+	+	+	+	+

+ indicates presence of homolog in the genome, - indicates absence of homolog in the genome, ++ indicates presence of 2 homologs in genome.

Genes encoding homologs to the core erythritol proteins EryA, EryB and EryD were ubiquitous throughout our data set (Table  2). With respect to the remaining genes, the genetic content of the species can be grouped into three broad categories. (1) Species that contain genes encoding homologs associated with erythritol, adonitol and L-arabitol catabolism. This includes *S. meliloti, S. medicae, S. fredii, M. loti, M. opportunism, M. ciceri, R. denitrificans* and *R. litoralis*. These genomes contained homologs to genes that encode enzymes specifically involved erythritol catabolism such as EryC, and TpiB as well as specifically involved in adonitol and L-arabitol catabolism including LalA*,* and RbtBC. They also contain genes encoding an ABC transporter homologous to the *S. meliloti* erythritol, adonitol and L-arabitol transporter (MptABCDE) and do not encode homologs to the *R. leguminosarum* erythritol transporter (EryEFG). One notable exception is *M. ciceri* which encodes EryEFG homologs rather than MptABCDE (Table  2). (2) Species that contain all the genes associated with erythritol catabolism, but lack the genes associated with adonitol or L-arabitol catabolism. These species include *R. leguminosarum* bvs. *viciae* and *trifolii*, *A. radiobacter, O. anthropi, B. suis*, *B. melitensis*, and *E. fergusonii*. These loci encode EryABCDR-TpiB as well as homologs to the *R. leguminosarum* ABC transporter EryEFG, but lack genes encoding homologs to enzymes associated specifically with adonitol and L-arabitol catabolism or the *S. meliloti* transport protein MptABCDE. *E. fergusonii* contains the most minimal set of homologs to erythritol genes of all the genomes investigated, and did not encode EryR and TpiB. (3) Species that do not encode the specifically erythritol associated EryC, EryR, and TpiB, but encode the adonitol/L-arabitol catabolic complement LalA-RbtABC and homologs to the *S. meliloti* polyol transporter MptABCDE. These include *Bradyrhizobium* spp. BTAi1 and ORS278, *A. multivorum, A. cryptum* and *V. eiseniae*.

### The genetic structure of erythritol loci

The genetic context of *eryA* in each of the genomes in our data set supported that each of these organisms contained an erythritol locus. A physical map of the loci in each of these organisms is depicted in Figure  1. Of note, a number of putative erythritol loci were identified in organisms with incomplete genome sequences at the time of analysis, and thus are not discussed here, including: *Octadecabacter antarcticus*, *Pelagibaca bermudensis Enterobacter hormaechei, Fulvimarina pelagi, Aurantimonas* sp. SI85-9A1, *Roseibium* sp. TrichSKD4, *Burkholderia thailandensis* and *Stappia aggregata.*

**Figure 2:**
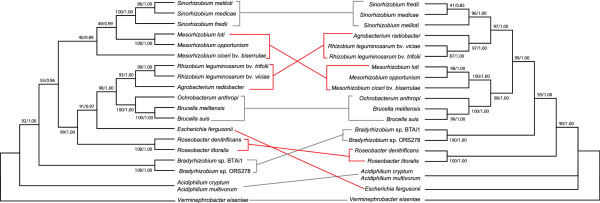
**The phylogenetic tree of erythritol proteins does not correlate with species phylogeny; evidence for horizontal gene transfer.** EryA phylogenetic tree (Left) and RpoD species tree (Right) were constructed using ML and Bayesian analysis. Support for each clade is expressed as a percentage (Bayesian/ML, ie. posterior probability and bootstrap values respectively) adjacent to the nodes that supports the monophyly of various clades. *V. eiseniae* was used as an outgroup for both trees since it was the most phylogenetically distant organism. A tree including branch lengths for EryA is included as Additional file 1: Figure S1.

The putative erythritol loci of bacteria in our data set ranged in genetic complexity with the loci from *S. meliloti* and *S. medicae* containing 17 different genes, to the simplest being the locus of *E. fergusonii*, which contained only two divergently transcribed operons that are homologous to the *eryEFG* and *eryABCD* loci of *R. leguminosarum*. A number of species contained loci that were identical in content and arrangement to the *R. leguminosarum* erythritol locus including members of the *Brucella*, *Ochrobacterum*, and *Agrobacterium*. The only species that contains a locus identical in content and arrangement to *S. meliloti* is the closely related *Sinorhizobium medicae*. The locus of *Sinorhizobium fredii* NGR234, contains all but one of the genes (*fucA1*) found in the other *Sinorhizobium* loci (Figure  2).

The loci of *Mesorhizobium* species were varied, however all three *Mesorhizobium* sp. contained an independent locus with homologs to *lalA* and *rbtBC* elsewhere in the genome (Figure  1). Interestingly, while *Mesorhizobium loti* and *Mesorhizobium opportunism* both contain transporters homologous to *mptABCDE*, *Mesorhizobium ciceri* bv. *biserrulae* contains a transporter homologous to *eryEFG.* This operon also contains the same hypothetical gene that is found at the beginning of the *R. leguminosarum eryEFG* transcript*.* The transporters however, are arranged in a manner similar to that seen in *S. meliloti* and the gene encoding the regulator *eryD*, is found ahead of the transporter genes, whereas in *R. leguminosarum* and *Brucella*, *eryD* is found following *eryC* (Figure  1). We also note that whereas *M. loti* and *M. opportunism* both contain a putative fructose 1,6 bis phosphate aldolase gene between the *eryR-tpiB-rpiB* operon and *eryC*, a homolog to this is also gene is found adjacent to the *rpiB* in *Brucella*.

*Bradyrhizobium* sp. BTAi1, and ORS278, *A. cryptum* and *V. eiseniae* all have similar genetic arrangement to that of *S. meliloti*, except that they do not contain a homolog to *eryC*, or an associated *eryR-tpiB-rpiB* operon. These loci also differ primarily in their arrangement of *lalA-rbtBC* (Figure  1)*.*

### The phylogenies of erythritol proteins do not correlate with species phylogeny

The DNA sequences of 16S *rDNA* (data not shown) as well as the amino acid sequences of RpoD were extracted from GenBank to analyze the phylogenetic relationships of the organisms examined in this study, using the most phylogenetically distant organism *Verminephrobacter eiseniae* as an out-group. The results of the 16S *rDNA* and RpoD sequence analyses were in concordance with each other and are consistent with phylogenies that have been previously generated [42]. Initial comparison of the operon structures with the generated phylogenies suggested that the operon structure(s) did not correlate with the species phylogeny. Since the structure of some operons did not correspond well with the species phylogenies we wished to determine if operon structure did correlate with any of the erythritol genes found at the *S. meliloti* loci. Since homologs to EryA, EryB and EryD were ubiquitous through the data set, it was decided to construct phylogenies based on Maximum Likelihood and Bayesian analysis using the EryA, EryB and EryD data sets. The topology of the phylogenetic tree using EryA is presented in Figure  2. A tree including branch lengths is included as Additional file 1: Figure S1. *V. eiseniae* was also the most distant member with respect to the EryA phylogeny and again used as an outgroup. The phylogenetic trees of EryB and EryD are not shown but were generally consistent with the EryA phylogeny. The species tree, based on RpoD, was included as a mirror tree with the EryA tree to demonstrate possible horizontal gene transfer events (Figure  2).

The data show that there is a high degree of correlation between the loci configuration and the EryA phylogenetic tree (Figure  1, 2). We note the similarity of the loci of *A. radiobacter* and *R. leguminosarum* to *Brucella* species and *O. anthropi* but not to the more closely related *Sinorhizobium* species. This suggests that a horizontal gene transfer may have occurred between these organisms. This is in agreement with what has been previously reported [20]. It also seems likely that a horizontal gene transfer event may have occurred between the *Brucella* and *E. fergusonii*. This may explain the unique occurrence of the loci’s presence in a member of the gamma-proteobacteria. Finally, our mirror tree suggests that a horizontal gene transfer of the more complex erythritol locus may have occurred between *M. loti* and an ancestral species the *Sinorhizobium* species (Figure  2)*.*

### Modes of evolution for the polyol utilization loci

Comparison of the phylogenetic trees of EryA, EryB and EryD to the arrangement and content of the loci led us to more thoroughly investigate the phylogenies of a number of proteins that stood out as unique within the data set. These phylogenies have led us to postulate modes of evolution that may have occurred in these loci.

**Figure 3:**
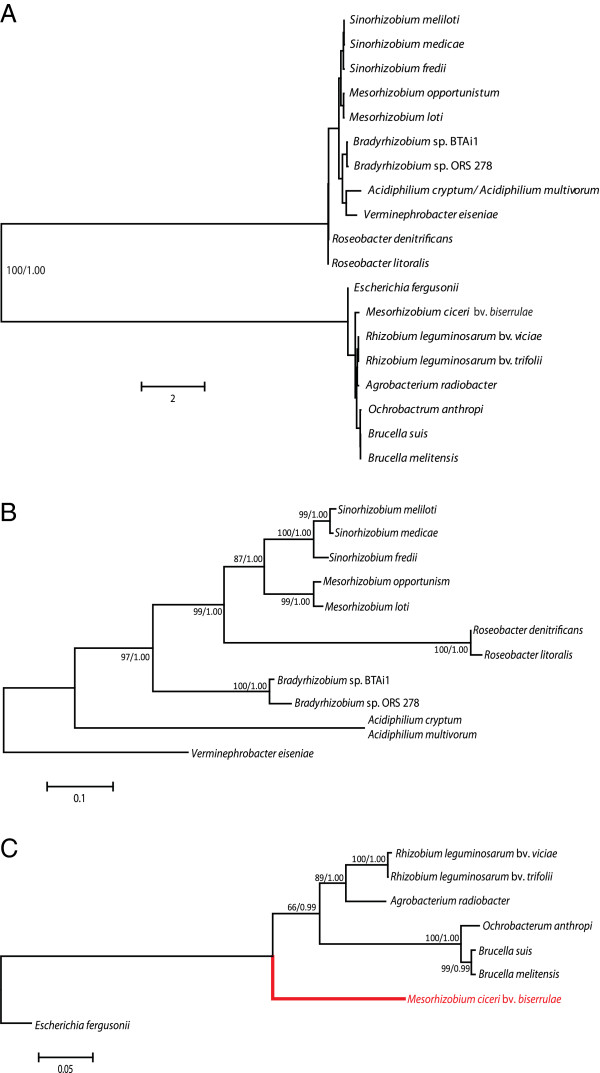
**Phylogenetic trees of erythritol transporters.** Unrooted phylogenetic tree including putative homologues to the sugar binding protein MptA of *Sinorhizobium meliloti* and EryG of *Rhizobium leguminosarum* (**A**). Support is provided for the node that clearly separates the putative homologues into two distinct and distant clades. Separate phylogenetic trees for erythritol transporters homologous to MptABCDE and EryEFG are depicted (**B** and **C**) using aligned amino acid sequences of the putative sugar binding proteins MptA (**B**) and EryG (**C**) as representatives of the transporters phylogenies. The branch that shows the anomalous placement of the *Mesorhizobium ciceri* bv. *biserrulae* within the tree of EryEFG homologs is highlighted in red. Trees were constructed using ML and Bayesian analysis. Support for each node is expressed as a percentage based on posterior probabilities (Bayesian analysis) and bootstrap values (ML). The branch lengths are based on ML analysis and are proportional to the number of substitutions per site.

BLASTP analysis showed a clear distinction between the type of transporter encoded by each of the loci and the remaining genetic content. In general, loci that contained adonitol/L-arabitol type genes contained a transporter homologous to the *S. meliloti* MptABCDE (Table  2, Figure  1). Loci that contained only erythritol genes contained a transporter homologous to the EryEFG of *R. leguminosarum.* One exception to this correlation was *M. ciceri* bv. *biserrulae* which contained a homologous transporter to EryEFG rather than MptABCDE. This is interesting because *M. ciceri* groups with the other *Mesorhizobia* in the EryABD trees. In order to analyze the evolution of these transporters more clearly, phylogenetic trees were constructed of homologs to EryG and homologs to MptA (Figure  3). In general the phylogenies are in agreement with the EryABD phylogenies, with the exception of *M. ciceri* which falls on a basal branch of the EryG phylogeny. The disparities between the EryG and EryABD phylogenies of *M ciceri* strongly suggest that parts of its erythritol locus have a different origin. This may have been the result of horizontal gene transfer of a second *R. leguminosarum* type erythritol locus, followed by recombination between the two.

**Figure 4:**
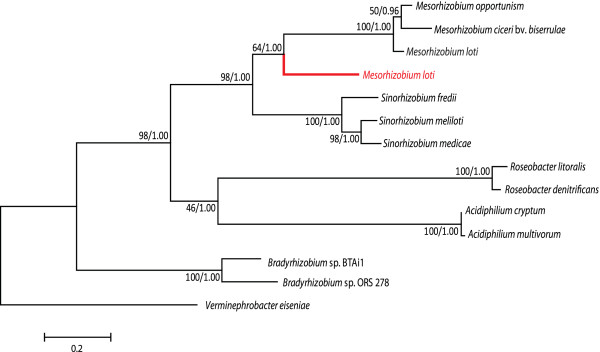
***Mesorhizobium loti *contains paralogs of LalA. ** The phylogeny of the L-arabitol catabolic gene LalA is depicted. *Mesorhizobium loti* contains a copy of *lalA* within an independent suboperon like the other *Mesorhizobium* species, as well as a second *lalA* homolog within the erythritol locus (Figure  1). The branch corresponding to the additional homolog within the erythritol locus is highlighted in red. The tree was constructed using ML and Bayesian analysis. Support for each node is expressed as a percentage based on posterior probabilities (Bayesian analysis) and bootstrap values (ML). The branch lengths are based on ML analysis and are proportional to the number of substitutions per site.

**Figure 5:**
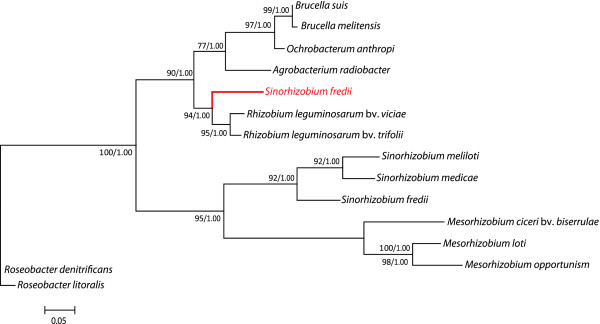
***Sinorhizobium fredii *encodes TpiB xenologs. ***Sinorhizobium fredii* contains a second suboperon that appears homologous to the *eryR-tpiB-rpiB* suboperon in the erythritol locus (Figure  1). The TpiB amino acid sequence was used as a representative of this suboperon to construct a phylogenetic tree. The branch corresponding to the TpiB encoded outside of the erythritol locus is highlighted in red. The tree was constructed using ML and Bayesian analysis. Support for each node is expressed as a percentage based on posterior probabilities (Bayesian analysis) and bootstrap values (ML). The branch lengths are based on ML analysis and are proportional to the number of substitutions per site.

In two organisms, apparent duplications of genes were present. In *M. loti* one homolog of *lalA* was present in the erythritol locus, while a second copy was present elsewhere in the genome adjacent to homologues of *rbtB* and *rbtC*, consistent with its location in the other two *Mesorhizobium* genomes. In *S. fredii* homologs to the apparent small operon that contains *eryR-tpiB-rpiB* were found both, as expected, in the erythritol locus, but also elsewhere on the chromosome in the same arrangement. To analyze the evolutionary history of these duplications phylogenetic trees were constructed for the LalA and TpiB homologs (Figure 4 and 5). The two copies of the *lalA* gene in *M. loti* are most likely an example of paralogs, as they still group within the same clade among other *lalA* homologs (Figure  4). The *tpiB* genes (Figure  5) in *S. fredii* are possible examples of xenologs [43] as the phylogenetic tree shows that the two versions of the *tpiB* gene in *S. fredii* are only distantly related, with one homolog grouping within the expected clade that includes S. *medicae* and *S. meliloti* and the second homolog (not part of the main locus) showing monophyly with those found in a clade containing *R. leguminosarum* sp., *B. suis*, etc. (Figure  5).

## Discussion

A number of models that are not mutually exclusive have been proposed to account for the formation and evolution of operons. Two broad aspects need to be considered, transfer of genes between organisms, as well as gathering and distributing genes within a genome. There is strong support for horizontal gene transfer as a driving force for evolution of gene clusters [44]. More recently, it has been shown that genes acquired by horizontal gene transfer events appear to evolve more quickly than genes that have arisen by gene duplication events [45]. Within a genome the “piece-wise” model suggests that complex operons can evolve through the independent clustering of smaller “sub-operons” due to selection pressures for the optimization for equimolarity and co-regulation of gene products [6]. Finally it has been suggested that the final stages of operon building can be the loss of “ORFan” genes [4,6].

The data presented here provide examples supporting these models of operon evolution. The components of the polyol catabolic loci we have identified have been involved in at least 3 horizontal gene transfers within the proteobacteria (Figure  2). In addition, components such as the transporter *eryEFG* have been moved from the *R. leguminosarum* clade of loci into the *M. ciceri* bv. *biserrulae* polyol locus (see Figure 3A and 3B). The later species based on its phylogenetic position and category of polyol locus (*S. meliloti*) would have been expected to contain the *mtpA* gene. The presence of possible paralogs of *lalA* (Figure  4) and the presence of *tpiB* xenologs (Figure  5) are also evidence for duplication and horizontal transfer events. Since *S. fredii* also contains a homolog to *tpiA* of *S. meliloti* (data not shown)*,* to our knowledge, this is the only example of an organism containing three triose-phosphate isomerases (Figure  2, Figure  5).

A striking example of a horizontal gene transfer and genetic rearrangement is exemplified by *M. ciceri* (Figure  1, Figure  2). It is likely that an exchange between *M. loti* and a common ancestor of *S. meliloti, S. medicae* and *S. fredii* NGR234 occurred. *M. loti* is located in the same clade as the *Brucella* and *O. anthropi* in the species tree (Figure 2). Despite this, *M. loti* contains many of the genes corresponding to the adonitol and L-arabitol type loci of other species that cluster close to the base of the species tree such as *Bradyrhizobium* spp. (Figure  2). The presence of these factors in addition to the chimeric composition of the *M. loti* locus leads us to hypothesise that an ancestor of *M. loti* may have contained both an erythritol locus like that of the *Brucella* as well as a polyol type locus like that seen in the *Bradyrhizobia, A. cryptum* and *V. eiseniae.*

The *lalA, rbtB, rbtC* suboperon appears to be the key component of the polyol locus in the *Bradyrhizobium* type loci (Figure  1). Among the 19 loci identified, these three genes can be linked into a suboperon, embedded within the main locus (eg. *R. litoralis*) or split among two transcriptional units (see *A*. *cryptum* or *V. eiseniae*). As well, the gene module (or suboperon) *eryR, tpiB- rpiB* is presumably found in all erythritol utilizing bacteria. The acquisition of this module along with the *lalA, rbtB* and *rbtC* suboperon may have allowed for the evolution of the more complex *S. meliloti* type locus (see Figure  2).

The absence of *fucA* in *S. fredii* NGR234 and *M. loti* appears to be an example of the loss of an “ORFan” gene event having occurred. The gene is still present in *S. meliloti* however it has been shown that it is not necessary for the catabolism of erythritol, adonitol, or L-arabitol [15]. It is likely that it was lost during the divergence of *M. loti* and *S. fredii* NGR234 from their common ancestors to *S. meliloti.* If this is true, it may be reasonable to assume that *fucA* may eventually also be lost from the *S. meliloti* erythritol locus.

In *S. meliloti*, erythritol uptake has been shown to be carried out by the proteins encoded by *mptABCDE*[15,16], whereas in *R. leguminosarum* growth using erythritol is dependent upon the *eryEFG*[20]. Although both transporters appear to carry out the same function, the phylogenetic analysis clearly shows that they have distinct ancestors and may be best classified as analogues rather than orthologues (Figure  3). In addition, it has been shown that MptABCDE is also capable of transporting adonitol and L-arabitol [15]. We note that these polyols appear to have stereo-chemical identity over three carbons and that EryA of *S. meliloti* can also use adonitol and L-arabitol as substrates [15]. It is unknown whether EryA from *R. leguminosarum* has the ability to interact with these substrates.

The three distinct groups of loci we have identified probably correspond to the metabolic potential of these regions to utilize polyols. The locus of *S. meliloti* has been shown to contain the full complement of genes required to confer growth on using both erythritol and adonitol and L-arabitol as sole carbon sources [15,16]. Given that *S. fredii* NGR234 and *M. loti* each contain homologs to all of these genes, except for *fucA* which is not necessary for the catabolism of any of the sugars [15], it follows that these two loci may also be capable of catabolising all three polyols. It has also been established that the *B. abortus* and *R. leguminosarum* type loci are used for erythritol catabolism, and given the annotation and degree of relatedness (E value = 0) of proteins belonging to all species in the clade, it is not expected that these loci would be capable of breaking down additional polyols [20,21]. This is supported by the fact that the introduction of the *R. leguminosarum* cosmid containing the erythritol locus into *S. meliloti* strains unable to utilize erythritol, adonitol, and L-arabitol were unable to be complemented for growth on adonitol and L-arabitol [15]. It is however necessary to remember that some of identified loci are only correlated with polyol utilization based on our analysis and that basic biological function, such as the ability to utilize these polyols has not been previously described.

With the advent of newer generations of sequencing technologies a greater number of bacterial genomes will be sequenced. It is likely that more examples of rearrangements of catabolic loci through bacterial lineages will be observed. Since the ability to catabolize erythritol is found in relatively few bacterial species, operons that encode erythritol and other associated polyols may be ideal models to observe operon evolution.

## Conclusions

In this work we show that there are at least three distinct erythritol/polyol loci arrangements. Two distinct ABC transporters can be found within these within these loci and phylogenetic analysis suggests these should be considered analogs. Finally we provide evidence that suggest that these loci have been horizontally transferred from the alpha-proteobacteria into both the beta and gamma-proteobacteria.

## Competing interests

The authors declare that they have no competing interests.

## Authors’ contribution

BAG collected the data set, performed the analysis and contributed to writing of the manuscript. GH provided advice and assistance with the analysis as well as contributed to the writing of the manuscript. IJO provided advice for the analysis and contributed to the writing of the manuscript. All authors read and approved the final manuscript.

## Supplementary Material

Additional file 1: Figure S1 EryA phylogenetic tree was constructed using ML and Bayesian analysis. Support for each clade is expressed as a percentage (Bayesian / ML, ie. posterior probability and bootstrap values respectively) adjacent to the nodes that supports the monophyly of various clades. The branch lengths are based on ML analysis and are proportional to the number of substitutions per site. This phylogenetic tree was used in the mirror tree in Figure 2 without branch lengths due to space restrictions. Click here for file
